# Special Issue: Diversity of Extremophiles in Time and Space

**DOI:** 10.3390/microorganisms9122472

**Published:** 2021-11-30

**Authors:** Fernando Puente-Sánchez, Max Chavarría

**Affiliations:** 1 Department of Aquatic Sciences and Assessment, Swedish University for Agricultural Sciences (SLU), Lennart Hjelms väg 9, 756 51 Uppsala, Sweden; 2 Escuela de Química, Universidad de Costa Rica, San José 11501-2060, Costa Rica; 3 Centro de Investigaciones en Productos Naturales (CIPRONA), Universidad de Costa Rica, San José 11501-2060, Costa Rica; 4 Centro Nacional de Innovaciones Biotecnológicas (CENIBiot), CeNAT-CONARE, San José 1174-1200, Costa Rica

Extreme environments are fascinating ecosystems that have allowed us to increase our knowledge about the evolutionary processes of life [1], develop new biotechnological applications (e.g., industrial applications of lipases [2], and thermostable DNA Polymerases in PCR tests [3]) and establish some fundamental concepts about the origins of life and the search for life in the Universe [1]. Despite the fact that research on the living beings that inhabit these extreme environments (i.e., extremophiles) began more than five decades ago with the pioneering works of Thomas D. Brock [4], nowadays, we still have a lot to learn about microbial diversity, and especially about the metabolism and biochemistry of these microorganisms; therefore, the study of extremophiles, extremozymes and their biotechnological potential remains a hot topic.

Over time, scientists have built the knowledge of extremophiles, overcoming intrinsic challenges such as the risks that are sometimes associated with sampling in extreme environments, the problems posed by the heterogeneous and dynamic nature of extreme ecosystems and the difficulties reproducing the physicochemical conditions required to cultivate these microorganisms [5,6,7]. With the development of new DNA sequencing methodologies in the 1990s, knowledge of the microbial diversity and biological processes in these environments increased exponentially; however, it also became evident that isolation was irreplaceable in order to fully characterize the physiology and behavior of microorganisms. Therefore, the development of new cultivation strategies continues to be imperative.

Extreme environments, generated by both natural and anthropogenic processes, are highly dynamic systems, which also reinforces the importance of a sustained study of the geomicrobiology of these environments over time. Great changes have been observed in both geochemistry and microbiology due to seasonality in different parts of the planet [8,9]. In addition, within these ecosystems, drastic changes in physicochemical conditions are usually generated over small distances (e.g., oxygen content and temperature [10,11]) leading to the formation of micro-niches colonized by specialized microbial communities. With this fact in mind, this Special Issue, “Diversity of Extremophiles in Time and Space”, presents a series of scientific articles dedicated to the study of extremophiles but focused on space–time variations in these extreme ecosystems.

The Special Issue starts with a paper by Procházková et al. [12] that presents a morphological, molecular and ecophysiological study of glacial algae from the European Alps. Specifically, the authors explored the similarities and differences between glacial algae, *Mesotaenium berggrenii* var. *alaskanum* (renamed as *Ancylonema alaskana* in this paper) and *Ancylonema nordenskioeldii*, present in samples taken at Morteratsch Glacier in Switzerland and Gurgler Ferner in Austria. These fascinating ecosystems are characterized by excessive irradiation, limited nutrients and very low temperatures, and constitute a challenge for photoautotrophic life. The authors, through a variety of techniques including morphological and molecular analyses, light-dependent performance, the analysis of phenols and lipidomics, concluded that *A. alaskana* is physiologically very similar and closely related, but genetically distinct, to *A. nordenskioeldii*. The relevance of this work is, in our opinion, twofold: first, it disproves the previous notion that *Ancylonema* was a monotypic genus; secondly, it shows that both known species of the genus have different photophysiologies, suggesting that *Ancylonema* has diversified in a way that allows it to exploit different light niches, presumably increasing its spatial distribution while minimizing the competition between *A. alaskana* and *A. nordenskioeldii*. Therefore, this work offers valuable information to understand the factors that determine the prevalence of dark pigmented algae in glaciers in Europe and around the world.

In the second article of this Special Issue, Fazlutdinova et al. [13] lead us to the Kamchatkan peninsula (Russia), one of the most active volcanic regions on Earth. There, the authors investigated the diversity of diatoms in volcano soils of the Mutnovsky and Gorely volcanoes. Samples from thirteen different soils were collected (including volcanic ash, rocks, humus–ocher soils, tundra volcanic illuvial–humus soils and sulfur deposits), identifying 38 different taxa. The genera *Pinnularia* and *Eunotia* were the most diverse in the studied area. The results of this study show the variations in diatom algae that can occur in volcanic environments in response to geochemical variations. Cosmopolitan diatoms are amongst the first microorganisms that colonize volcanic substrates after an eruption, and they were found, accordingly, in the samples closest to the volcano craters. The number of diatom species increased with the distance to the crater, in parallel with the development of moisture, nutrients and vegetation. Soil alkalinity also favored a higher diatom diversity. Overall, this is a fine example of how diatom populations change in time and space as volcanic soils undergo different successional stages. 

Continuing on the Kamchatkan peninsula (Russia), Frolov et al. [14] studied the diversity of sulfate-reducing prokaryotes (SRP) in the hot springs of Uzon Caldera, Mutnovskii Volcano and Geyser Valley. Samples from fifteen hot springs were analyzed, with temperatures ranging from 52 to 90 °C, pH levels between 2.5 and 6.6 and sulfate concentrations between 0.2 and 9.9 mM. The authors report variations in SRP diversity in these sites as a response to pH. In neutral and slightly acidic hot springs, the presence of *Desulfobacterota*, *Nitrospirota* and *Firmicutes* were identified, while the bacteria of phylum *Thermodesulofobiota* were identified in acidic hot springs. It is also reported that in high-temperature acidic springs, sulfate reduction was mediated by the archaea of the phylum *Crenarchaeota*. In general, SRP were involved in the final stages of organic matter degradation in the anaerobic niches, but different actors were active in the different samples. The great diversity of SRP thus ensured that this important ecosystem service remained operative regardless of the temperature or pH. 

Finally, Zgonik et al. [15] wrote a systematic review on the diversity and biotechnological potential of microorganisms present in different ecosystems in Central Europe including caves, ice caves, glaciers, cold depressions in shady areas of mountains, salterns and thermal and mineral springs. The different ecosystems discussed in this review present unique physicochemical characteristics and are inhabited by specialized microbial communities that have undoubtedly adapted to these conditions. The variety of environments, microbial diversity and biotechnological potential reviewed by Zgonik et al. [15] reinforces the importance of continuing with the investigation of extreme microorganisms, mainly in environments that have not been as explored, such as caves. Furthermore, the thorough identification of more than one hundred different sampling points in Central Europe, together with their GPS coordinates and general characteristics, will undoubtedly become an invaluable resource for microbial ecologists seeking to design large-scale comparative studies.

This Special Issue compiles some of the work that is being conducted around the world in the field of extremophiles, with an emphasis on variations in microbial communities in response to spatio-temporal changes. Much remains for us to learn and continue investigating in this exciting area of research where complexity predominates and requires all our ingenuity and the best technological tools available to break down and understand this complexity.
